# The cucumber mosaic virus 1a protein regulates interactions between the 2b protein and ARGONAUTE 1 while maintaining the silencing suppressor activity of the 2b protein

**DOI:** 10.1371/journal.ppat.1009125

**Published:** 2020-12-03

**Authors:** Lewis G. Watt, Sam Crawshaw, Sun-Ju Rhee, Alex M. Murphy, Tomás Canto, John P. Carr

**Affiliations:** 1 Department of Plant Sciences, University of Cambridge, Cambridge, United Kingdom; 2 Department of Microbial and Plant Biotechnology, Center for Biological Research, Madrid, Spain; The Ohio State University, UNITED STATES

## Abstract

The cucumber mosaic virus (CMV) 2b viral suppressor of RNA silencing (VSR) is a potent counter-defense and pathogenicity factor that inhibits antiviral silencing by titration of short double-stranded RNAs. It also disrupts microRNA-mediated regulation of host gene expression by binding ARGONAUTE 1 (AGO1). But in *Arabidopsis thaliana* complete inhibition of AGO1 is counterproductive to CMV since this triggers another layer of antiviral silencing mediated by AGO2, de-represses strong resistance against aphids (the insect vectors of CMV), and exacerbates symptoms. Using confocal laser scanning microscopy, bimolecular fluorescence complementation, and co-immunoprecipitation assays we found that the CMV 1a protein, a component of the viral replicase complex, regulates the 2b-AGO1 interaction. By binding 2b protein molecules and sequestering them in P-bodies, the 1a protein limits the proportion of 2b protein molecules available to bind AGO1, which ameliorates 2b-induced disease symptoms, and moderates induction of resistance to CMV and to its aphid vector. However, the 1a protein-2b protein interaction does not inhibit the ability of the 2b protein to inhibit silencing of reporter gene expression in agroinfiltration assays. The interaction between the CMV 1a and 2b proteins represents a novel regulatory system in which specific functions of a VSR are selectively modulated by another viral protein. The finding also provides a mechanism that explains how CMV, and possibly other viruses, modulates symptom induction and manipulates host-vector interactions.

## Author summary

Cucumber mosaic virus (CMV) causes disease in over a thousand plant species including many crops. Aphids, insects with probing mouthparts that introduce virus particles directly into host cells, transmit CMV between plants. The 2b protein is the smallest protein encoded by CMV but has multiple functions. The 2b protein can disrupt the host RNA silencing pathways. These comprise mechanisms that regulate host gene expression (using microRNAs) and that degrade viral RNA molecules (using short-interfering RNAs). The 2b protein can bind to short-interfering RNAs to inhibit host resistance to virus infection and it can also bind the host protein Argonaute 1, disrupting microRNA-regulated host gene expression. However, in *Arabidopsis thaliana* excessive inhibition of Argonaute 1 activity may trigger excessive damage to the host, enhance CMV resistance, and render plants toxic to aphids, which may be deleterious to virus transmission. We found that another CMV protein, the 1a protein (a component of the CMV replication machinery), binds to 2b protein molecules and limits 2b-Argonaute 1 interactions without causing any detectable impairment of the ability of the 2b protein to counteract antiviral RNA silencing. This allows CMV to inhibit resistance against itself and its vectors, while preventing excessive damage to its host plant.

## Introduction

Cucumber mosaic virus (CMV) is agronomically important and has one of the largest viral host ranges (>1,000 species) [1]. CMV has a tripartite positive-sense RNA genome [2,3]. RNA1 encodes the 110kDa 1a protein, which has methyltransferase and helicase activity, and forms part of the viral replicase complex [4–7]. The 1a protein also influences viral systemic movement and symptom severity [8–10]. RNA2 has two open reading frames (ORFs). The 5’-proximal ORF of RNA2 encodes another replicase complex component, the 97 kDa 2a protein, which has RNA-dependent RNA polymerase activity [4–7]. The 3’-proximal ORF of RNA2 encodes the 2b protein, which has a predicted mass of 12–13 kDa but migrates with an apparent mass of *c*. 17 kDa in sodium dodecyl sulfate-polyacrylamide gel electrophoresis (SDS-PAGE) [11]. The 2b protein is multifunctional but is best known as a viral suppressor of RNA silencing (VSR) [2,12]. RNA silencing is an important antiviral mechanism but the 2b protein also disrupts resistance mediated by the defense and stress-related phytohormones salicylate, jasmonate, and abscisic acid; the latter two also being important signals in defense against insects [13–19]. CMV RNA3 has two ORFs that, respectively, encode the movement and coat proteins [2,3].

Taxonomically, *Cucumber mosaic virus* is the type species of the genus *Cucumovirus* in the family *Bromoviridae* [1]. Based on RNA sequence data most CMV strains can be classified into two major Subgroups (I and II), and Subgroup I strains can be further assigned to Subgroups IA and IB [2,3,20]. Within Subgroups, the 2b protein amino acid sequences are highly conserved [11,21]. Although all CMV 2b proteins can accumulate in the host cell nucleus, the 2b proteins encoded by CMV strains in Subgroups IA and IB also associate with the nucleolus, cytoplasm and cytoskeleton [11,21–23].

The VSR activity of cucumoviral 2b proteins arises predominantly from binding double-stranded (ds) short-interfering RNAs [siRNAs], which requires 2b dimer or tetramer formation *in vivo* [24–28]. However, 2b proteins from Subgroup I also bind ARGONAUTE (AGO) proteins 1 and 4 [23,25,26,29,30]. CMV-induced developmental symptoms are conditioned partly through 2b-AGO1 interactions and consequent interference with microRNA (miRNA)-regulated gene expression [30–33], and via unknown effects that 2b proteins exert in the nucleus [22].

Aphids of over 80 species vector CMV in the non-persistent manner, i.e., virions bind to receptors within the aphid stylet and are acquired and lost rapidly during short probes of plant epidermal cells [34,35]. Rapid, local transmission is most efficient when aphids alight briefly on infected plants, sample the epidermal cell contents and disperse [36–38]. However, epidemiological modeling indicates that while rejection of a host following a brief sampling feed encourages rapid localized virus transmission by wingless aphids, settlement and reproduction of aphids on plants will eventually favor longer distance virus dissemination by winged aphids [36].

CMV seems to be able to manipulate host-aphid interactions to promote its own transmission [36–41]. The effects of CMV on plant-aphid interactions are host-specific. For example, in squash (*Cucurbita pepo*) and tobacco (*Nicotiana tabacum*) the Subgroup IA CMV strain Fny (Fny-CMV) induces changes in the emission of plant volatile organic compounds (VOCs) and those produced by infected cucurbits have been shown to influence aphid foraging behavior [42–44]. Fny-CMV induces production of distasteful substances (antixenosis) in squash and *Arabidopsis thaliana* plants. Antixenosis promotes virus acquisition from epidermal cells, inhibits phloem feeding, and promotes aphid dispersal [36,41,43].

The 2b protein influences host-aphid interactions [18,40,41,45]. In tobacco, the mutant virus CMVΔ2b (which cannot express the 2b protein) induces strong anti-aphid resistance (antibiosis) that increases the mortality of aphids (*Myzus persicae*) [40]. In tobacco the 1a protein is the factor that triggers antibiosis, but during infection with wild-type CMV induction of antibiosis (which is deleterious to aphid-mediated transmission) is counteracted by the 2b protein [40,46]. However, the Fny-CMV 2b protein appears to have the opposite effect in Arabidopsis. Constitutive expression of the Fny-CMV 2b protein in transgenic Arabidopsis plants induces antibiosis [41]. Since Arabidopsis AGO1 negatively regulates antibiosis against aphids, it was concluded that 2b-induced antibiosis results from the interaction of the Fny-CMV 2b protein with AGO1 [41,47]. However, the 2b protein is not responsible for the antixenosis induced in Arabidopsis by Fny-CMV infection [41]. Instead, the Fny-CMV 2a protein triggers increased biosynthesis of methoxy-indol-3-yl-methylglucosinolate, which discourages aphids from prolonged phloem feeding [41].

How is 2b-induced antibiosis prevented during CMV infection of Arabidopsis? Co-expression of 1a and 2b proteins in transgenic plants inhibited aphid resistance and also ameliorated the 2b-induced developmental abnormalities that occur in *2b*-transgenic Arabidopsis plants [32,41]. This suggested that the CMV 1a protein negatively regulates the ability of the 2b protein to inhibit AGO1 activity [41]. We investigated if the 1a protein inhibits 2b-AGO1 interactions indirectly or by directly interacting with either the 2b protein or AGO1, and if these interactions affect the VSR activity of the 2b protein, or just its ability to interact with AGO1.

## Results

### The CMV 1a protein inhibits 2b-induced resistance to aphid colony growth

**Fig 1:**
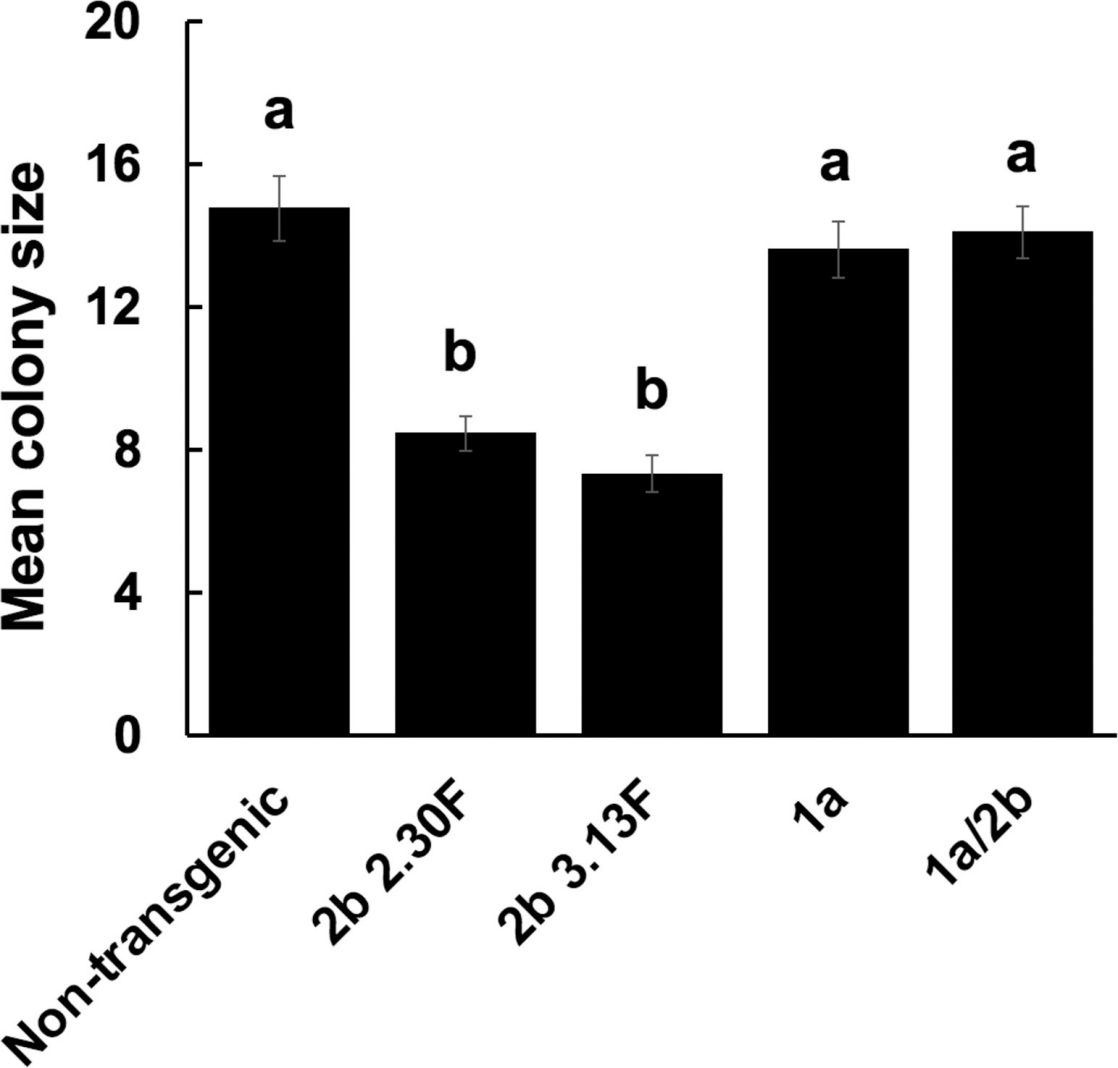
The effects on aphid reproduction of cucumber mosaic virus (CMV) infection and transgenic expression the CMV 1a and 2b proteins in *Arabidopsis thaliana*. Individual one-day-old *Myzus persicae* nymphs (n≥16) were placed on plants and number of offspring (colony size) counted at 10 days post-infestation. Aphids were placed on plants that were: non-transgenic or transgenic plants constitutively expressing the CMV 2b protein (lines 2.30F and 3.13F [32]), the CMV 1a protein or both the 1a and 2b proteins [41]. Different letters are assigned to significantly different groups (ANOVA with *post-hoc* Tukey’s tests, *P*<0.05). Error bars represent standard error of the mean.

Westwood and colleagues [41] showed that the growth of aphids (*M*. *persicae*) confined on *2b*-transgenic plants is inhibited but that this does not occur on doubly transformed *1a/2b*-transgenic plants. We observed that another aphid performance indicator (aphid fecundity) was also affected on transgenic plants expressing the 2b protein (Fig 1). Aphid colony growth on *2b*-transgenic plants was significantly decreased compared to that on non-transgenic plants but no reduction of colony growth occurred on *1a*-transgenic plants or on double *1a/2b*-transgenic plants (Fig 1). This shows that 2b-induced antibiosis affects not only the growth of individual aphids but also their ability to reproduce, and that the CMV 1a can counter both 2b-induced aphid resistance phenomena. These observations led us to investigate the possibility that the 1a and 2b proteins interact with each other either directly, or indirectly, for example by competing for binding to a cellular factor such as AGO1.

### Subcellular localization of 1a and 2b CMV proteins

**Fig 2:**
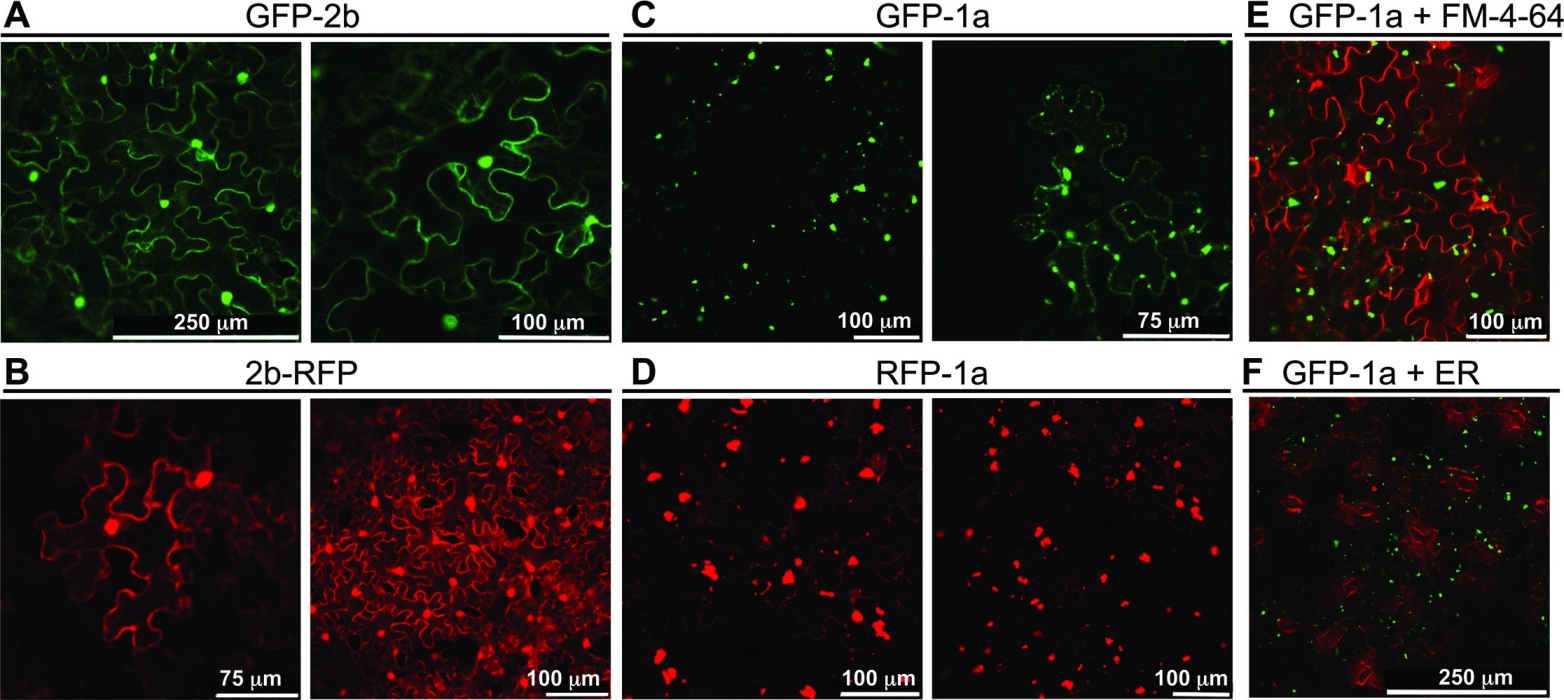
Sub-cellular localization of the cucumber mosaic virus 1a and 2b proteins. The GFP-1a and RFP-1a protein fusions were expressed in *N*. *benthamiana* leaves by agroinfiltration and images recorded 3–4 days later by confocal laser scanning microscopy. Consistent with previous investigations of the Fny-CMV 2b protein [22,23], GFP-2b (A) and 2b-RFP (B) accumulated in the nuclei and cytoplasm. In contrast, GFP-1a (C) and RFP-1a (D) accumulated as punctate specks of varying size. At higher magnification (C, right panel) GFP-1a accumulation at the cell periphery could be observed. However, staining with a membrane-binding dye (FM-4-64) indicated that the larger GFP-1a aggregations did not co-localize with the cell membrane (E). Staining with ER-tracker (ER) did not indicate co-localization of GFP-1a with the endoplasmic reticulum network (F).

To determine if a direct 1a-2b protein-protein interaction was likely, we studied the subcellular distribution of proteins comprising the 2b or 1a sequences fused with either green fluorescent protein (GFP) or red fluorescent protein (RFP) sequences. Fusion proteins were expressed transiently in *N*. *benthamiana* leaves by agroinfiltration, and fluorescence was imaged by confocal laser scanning microscopy. The 2b-RFP was generated by fusing the 2b protein C terminus with RFP, and GFP-2b by fusion of GFP to the N terminus of the 2b protein. Consistent with previous investigations [23], 2b-RFP and GFP-2b accumulated in the nuclei and cytoplasm (Fig 2A and 2B). Fluorescently tagged versions of the 1a protein were made with N-terminal fusions with either RFP (RFP-1a) or GFP (GFP-1a). Both RFP-1a and GFP-1a aggregated as punctate ‘specks’ (Fig 2C and 2D). These specks consisted of individual foci that also clustered to form larger aggregations. Some of the the 1a protein aggregates, as well as the smaller 1a protein foci appeared to be localized in proximity of the cell membrane (Fig 2C, left panel).

To determine if the 1a protein associated with intracellular membranes, the styryl membrane-binding dye FM-4-64 was used to stain leaf tissue agroinfiltrated with GFP-1a. Despite the fact that in several experiments, GFP-1a foci were observed close to the cell membrane, there was no strong indication of co-localization between the larger 1a protein aggregates and FM-4-64 dye (Fig 2E). To determine if larger GFP-1a aggregations corresponded to ER-derived vesicles, leaves agroinfiltrated with GFP-1a were stained with the dye ER-tracker (Fig 2E). No co-localization was observed between the GFP-1a and ER-tracker, indicating that the 1a protein aggregations are not localized to ER-derived vesicles.

**Fig 3:**
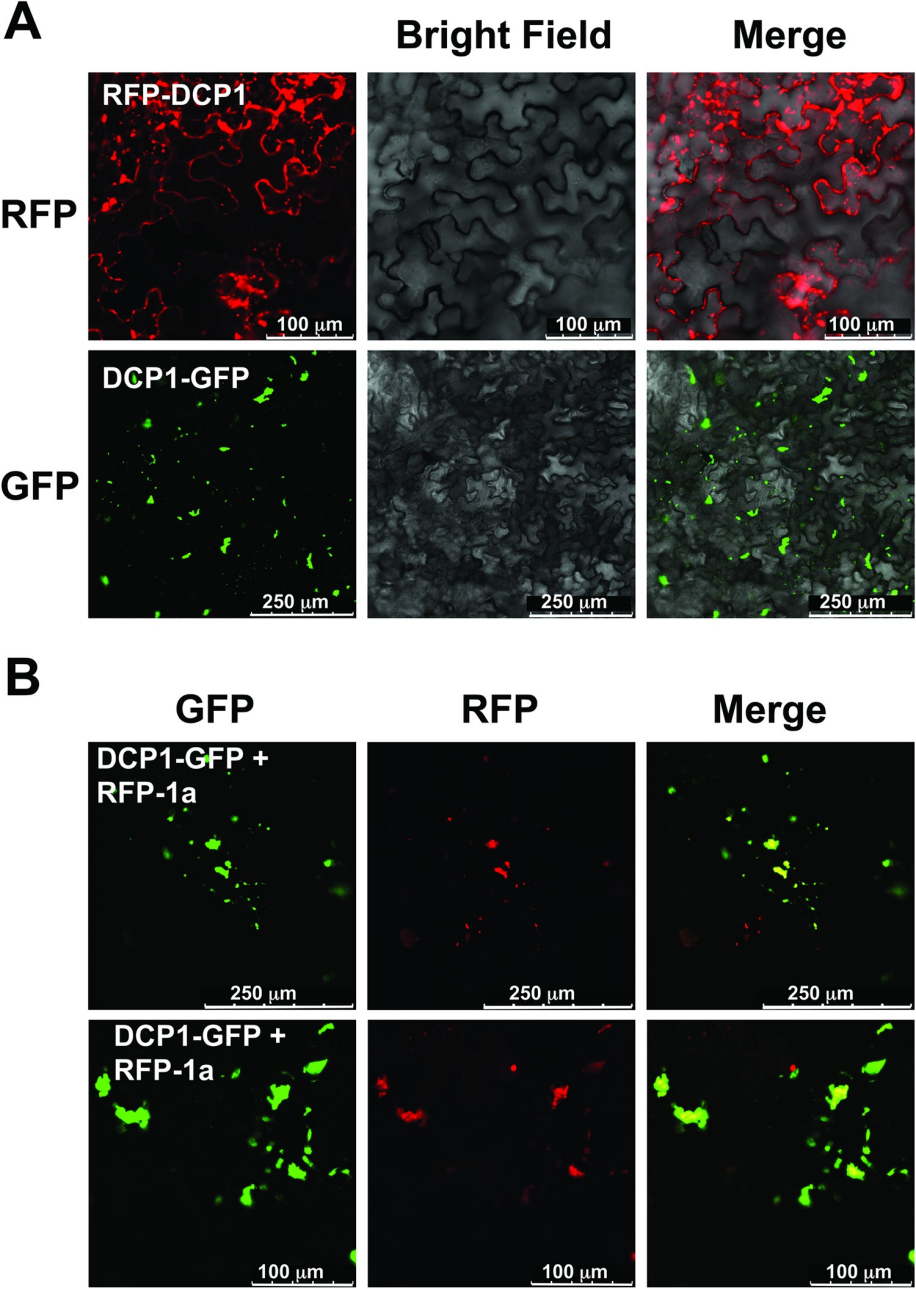
The P-body marker DCP1 localizes with the cucumber mosaic virus 1a protein. A. Fluorescence from DCP1-GFP was observed as punctate specks with varying size. The localization pattern of DCP1-GFP resembled that of RFP-1a, leading us to speculate that they may occupy the same subcellular compartment. RFP-DCP1 and DCP1-GFP were observed in small foci associated with the periphery of the cell, presumably P-bodies. DCP1-GFP fluorescence was brighter than DCP1-RFP so was used for co-localization experiments with RFP-1a (Panel B). B. When RFP-1a was co-agroinfiltrated with DCP1-GFP we observed co-localization of the two proteins in some of the specks.

The orthologous 1a protein of brome mosaic virus (BMV) associates with cytoplasmic processing bodies (P-bodies) [48]. We hypothesized that the CMV 1a protein also associates with P-bodies, which would be consistent with the punctate distribution of the 1a protein (Fig 2C and 2D). *A*. *tumefaciens* cells harboring the RFP-1a construct were co-agroinfiltrated with cells carrying a construct encoding the P-body marker protein DCP1-GFP. When infiltrated individually, RFP-DCP1 and DCP1-GFP formed punctate specks (Fig 3A). When DCP1-GFP was co-agroinfiltrated with RFP-1a the two proteins were observed to strongly co-localize (Fig 3B). Thus, a large portion of RFP-1a protein associated with P-bodies.

### The 1a protein interacts directly with the 2b protein but not with AGO1 in bimolecular fluorescence complementation assays

**Fig 4:**
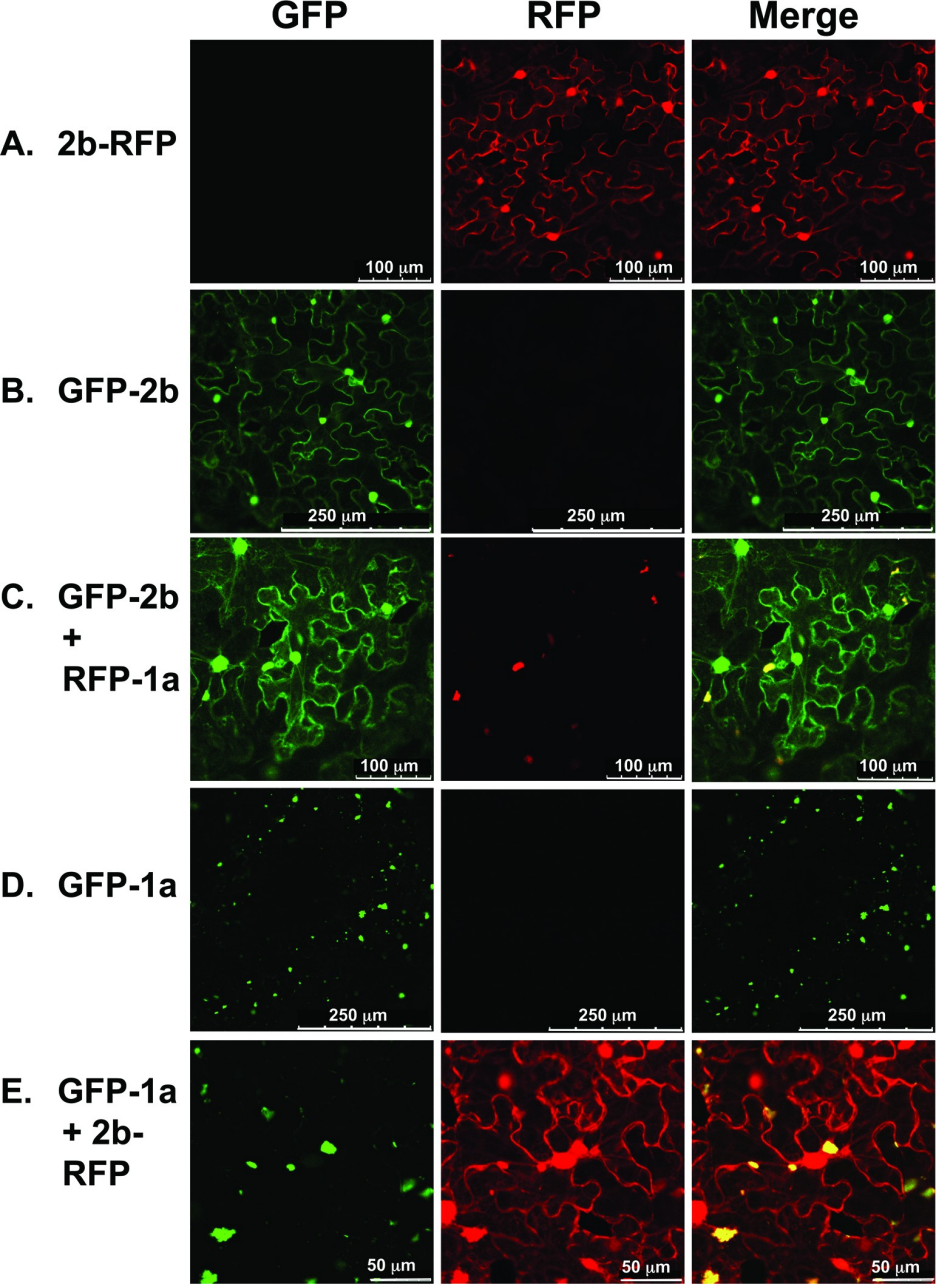
Subcellular localization of cucumber mosaic virus 1a and 2b proteins co-expressed transiently by agroinfiltration. A, B, fluorescence derived from CMV 2b protein tagged at its N-terminus with GFP or C-terminus with RFP. C, fluorescence originating from RFP-1a proteins accumulates at small ‘specks’ throughout the cytoplasm and as larger aggregates. Fluorescence originating from the GFP-2B proteins accumulated at the nucleus and evenly throughout the cytoplasm, as seen in panel B, but a portion of the signal was observed to be present in the same cellular compartment as RFP-1a signal yielding a merged signal shown as yellow. D, 1a protein tagged at its N-terminus with GFP expressed alone appeared as aggregates and smaller foci. E, fluorescence derived from CMV 2b protein tagged at its C-terminus with RFP and 1a protein tagged at its N-terminus with GFP. The 2b-RFP protein can be observed in the nucleus and cytoplasm, but additionally in specks that strongly co-localize with GFP-1a. This pattern of 1a and 2b co-localization is similar to C suggesting that the localization of 1a and 2b proteins is not biased by the presence of either GFP or RFP sequences.

To assess if localization of 2b or 1a is altered when both viral proteins are present *in vivo*, 2b and 1a proteins with different fluorescent tags were co-agroinfiltrated into *N*. *benthamiana* leaves. When agroinfiltrated singly, fluorescence due to GFP-2b or 2b-RFP proteins accumulated in the cytoplasm and nucleus (Fig 4A and 4B). However, when co-expressed with 1a protein, the fluorescent 2b proteins also co-localized to the fluorescent ‘specks’ (Fig 4C and 4E) observed for GFP-1a protein localization (Fig 4D). When the RFP-1a construct was co-agroinfiltrated with a construct encoding free, unfused GFP (35S:GFP), we did not observe re-localization of GFP to the sites where RFP-1a fluorescence accumulated (S1 Fig). Thus, 1a-2b co-localization is specific and does not occur as a result of non-specific binding of GFP to the 1a protein.

**Fig 5:**
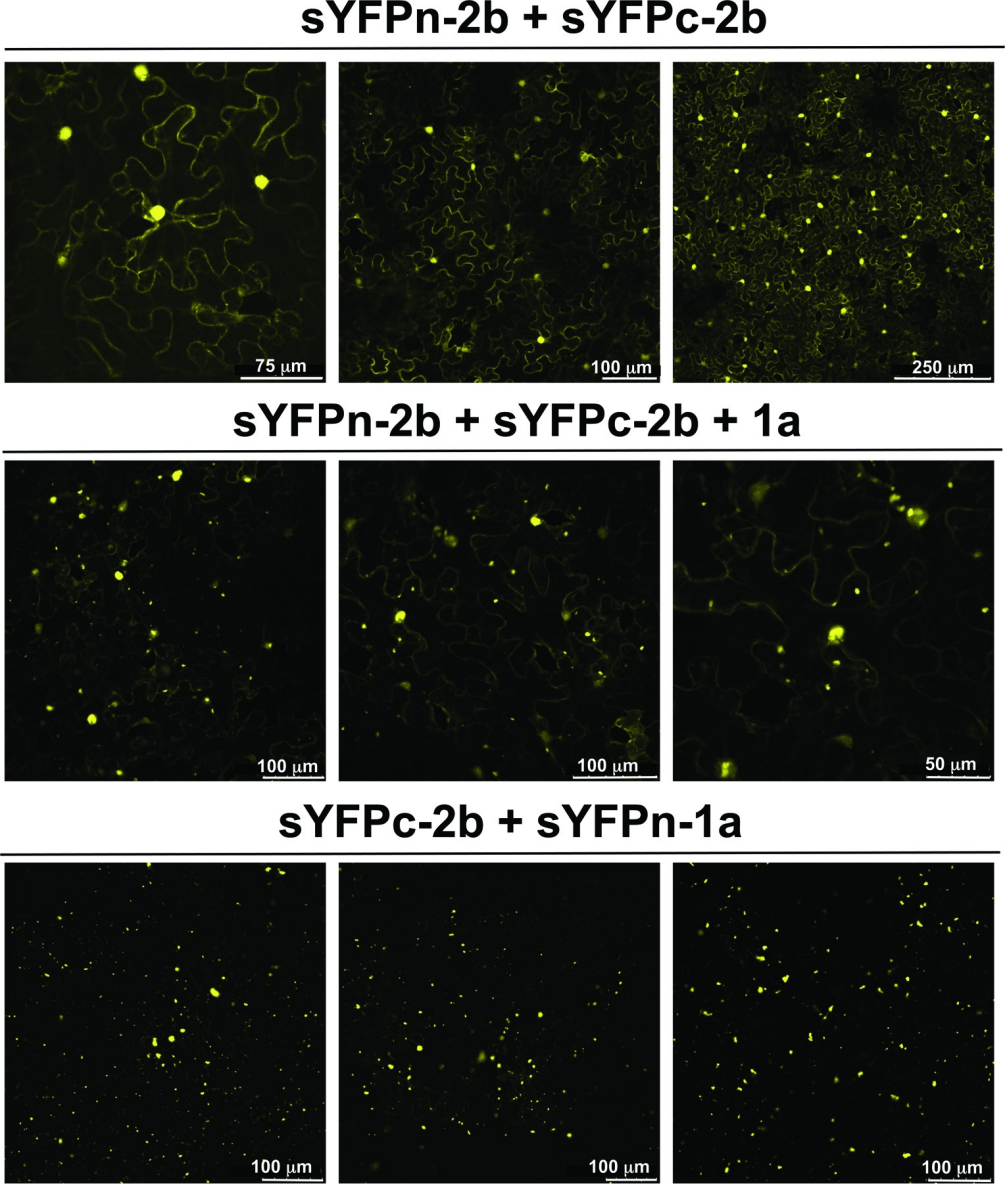
The cucumber mosaic virus 1a and 2b proteins both exhibit self-interaction and interact with each other *in planta*. The 2b and 1a proteins were tagged at their N-termini with split yellow fluorescent protein (sYFP) (sYFPn-2b, sYFPc-2b, sYFPn-1a, and sYFPc-1a) to study protein-protein interactions *in vivo* by bimolecular fluorescence complementation. When sYFPn-2b and sYFPc-2b were co-expressed transiently in *N*. *benthamiana* leaves, the observed pattern of fluorescence showed mainly nuclear localization, but also presence in the cytoplasm (upper three panels). When sYFPn-2b and sYFPc-2b were co-expressed with untagged 1a protein, the observed pattern of fluorescence, originating from the interaction of sYFP-2b proteins, still localized to the nucleus and diffusely in cytoplasm however, there was an additional pattern of fluorescence observed as specks within the cytoplasm (middle three panels). This suggests that the presence of 1a alters the localization of interacting sYFP-2b pairs possibly causing them to co-localize with the 1a protein. When sYFPn-1a and sYFPc-2b were co-expressed, a strong fluorescent signal was observed, which localized to distinct punctate specks within the cytoplasm, this pattern of localization was similar to that observed with GFP-1a and RFP-1a (lower three panels).

To visualize potential protein-protein interactions *in planta*, 2b, 1a and Arabidopsis AGO1 protein-coding sequences were fused with sequences encoding the yellow fluorescent protein (YFP) split into the N- and C-terminal portions (sYFPn and sYFPc, respectively) for bimolecular fluorescence complementation (BiFC) assays. Fluorescence derived from the reconstitution of the YFP fluorophore after sYFPn-2b and sYFPc-2b self-interaction localized to the nucleus and cytoplasm (Fig 5A). This pattern of fluorescence was similar to that observed with GFP-2b and 2b-RFP (Fig 2A and 2B) and consistent with previous studies using sYFP-2b [23]. The distribution of fluorescence for sYFP-2b changed following co-agroinfiltration of untagged 1a (Fig 5B) with fluorescence still visible in the nucleus and cytoplasm, but was additionally present at the ‘specks’ previously observed with GFP/RFP-1a expression (Fig 2C and 2D). It was previously shown that the N-terminal regions of the CMV and BMV 1a proteins self-interact [49]. We confirmed self-interaction for the CMV 1a protein (S2 Fig), although the fluorescence intensity was not as great as for the 2b-2b self-interaction.

BiFC with sYFPn-1a with sYFPc-2b constructs was used to determine if the 1a and 2b proteins interact directly. Strong fluorescence was observed that localized as ‘specks’ (Fig 5C), showing a similar pattern of fluorescence to that seen with GFP-1a and RFP-1a (Fig 2C and 2D). No fluorescence was observed when the sYFP halves were swapped at the N-terminal of the 1a and 2b fusion proteins, suggesting that the interaction of 2b with 1a reconstitutes the YFP protein only in certain conformations. When sYFP-1a and sYFP-AGO1 constructs were co-agroinfiltrated into *N*. *benthamiana* leaves no fluorescence was observed, indicating there is no direct 1a protein-AGO1 interaction *in planta* (S2 Fig). The 1a-2a protein interaction is known to be required for formation of an active replicase complex [49,50]. As an additional control, we confirmed the 1a-2a interaction by co-agroinfiltration of sYFP-1a and sYFP-2a constructs, which resulted in observable fluorescence that was localized to regularly sized small foci (S2 Fig).

**Fig 6:**
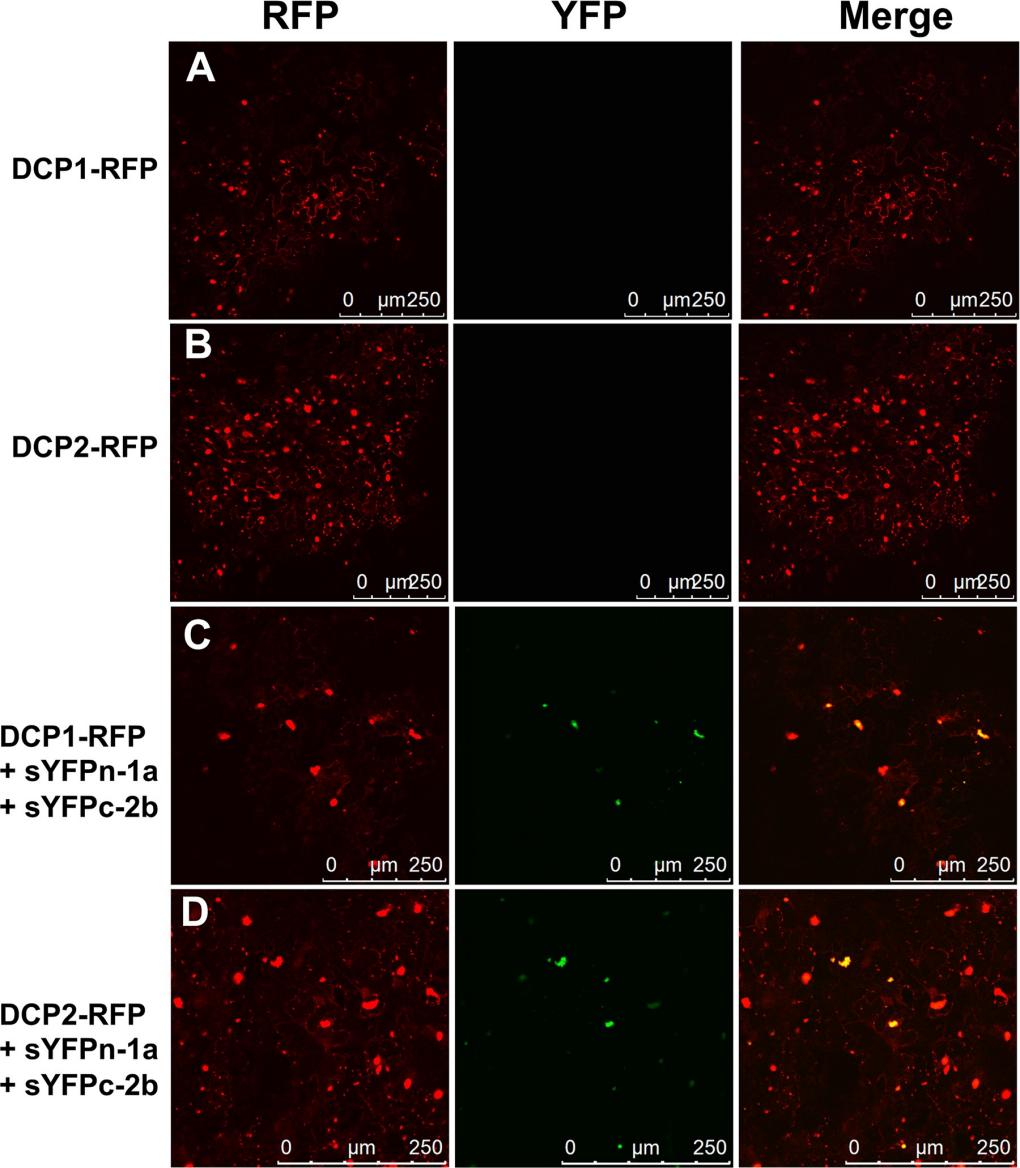
Complexes of the cucumber mosaic virus 1a and 2b proteins co-localize with two P-body markers. Constructs encoding either of the fluorescently tagged P-body marker proteins DCP1-RFP or DCP2-RFP [76] were expressed in *N*. *benthamiana* leaves by agroinfiltration either alone (A, B), or in leaves co-infiltrated with split YFP fusions for the CMV 1a and 2b proteins (C, D). Confocal laser scanning microscopy was used to observe the localization of DCP1-RFP and DCP2-RFP, and the YFP signal from complexes formed between sYFPn-1a and sYFPc-2b. To facilitate visualization of the localization of the DCP1-RFP and DCP2-RFP P-body markers versus that of sYFPn-1a-sYFPc-2b complexes in merged images, the YFP fluorescence signals have been false-colored green. sYFPn-1a-sYFPc-2b complexes localized exclusively to P-bodies.

The re-localization of the 2b protein by the 1a protein to P-bodies was further confirmed using BiFC combined with imaging of the P-body marker DCP1-RFP, and an additional P-body marker DCP2-RFP (Fig 6). Leaf tissue was co-infiltrated with *A*. *tumefaciens* cells harboring plasmids encoding either sYFPn-1a or sYFPc-2b, as well as cells carrying plasmids encoding either DCP1-RFP or DCP2-RFP. Confocal scanning laser microscopy of agroinfiltrated tissues confirmed that DCP1-RFP and DCP2-RFP accumulated in discrete specks, as expected (Fig 6A and 6B). Complexes of the 1a and 2b protein revealed by BiFC were observed to co-localize with both DCP1-RFP (Fig 6C), and with DCP2-RFP (Fig 6D). This further supports our finding that the 1a protein can complex with the 2b protein and that it re-allocates this portion of 2b protein pool to P-bodies.

### Co-immunoprecipitation of the 1a and 2b proteins

**Fig 7:**
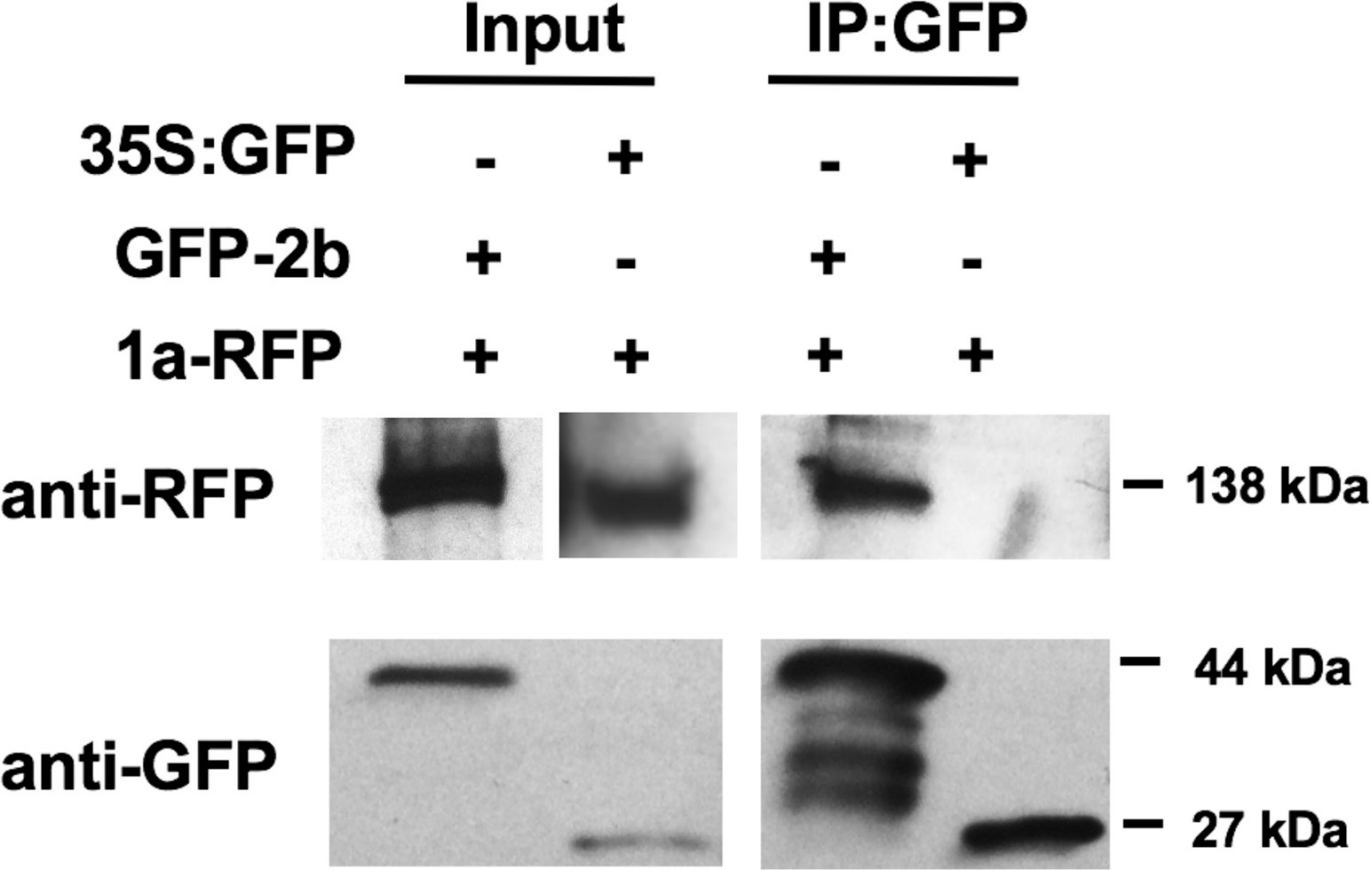
Association of the cucumber mosaic virus 1a and 2b proteins *in planta* demonstrated by co-immunoprecipitation. Total proteins from *N*. *benthamiana* leaves were subjected to immunoprecipitation with GFP-Trap beads followed by immunoblot analysis with anti-GFP antibodies to detect GFP-2b or 35S:GFP and anti-RFP antibodies to detect RFP-1a. RFP-1a could be detected in both input samples with a corresponding band of approximately 138kDa. After Immunoprecipitation with GFP-pull down RFP-1a could only be detected when co-expressed with GFP-2b, and was not detected with expressed with 35S:GFP. Imaged bands displayed are from the same blot but exposed to X-ray film for different periods for clarity. Original blots are shown in S4 Fig for comparison.

**Fig 8:**
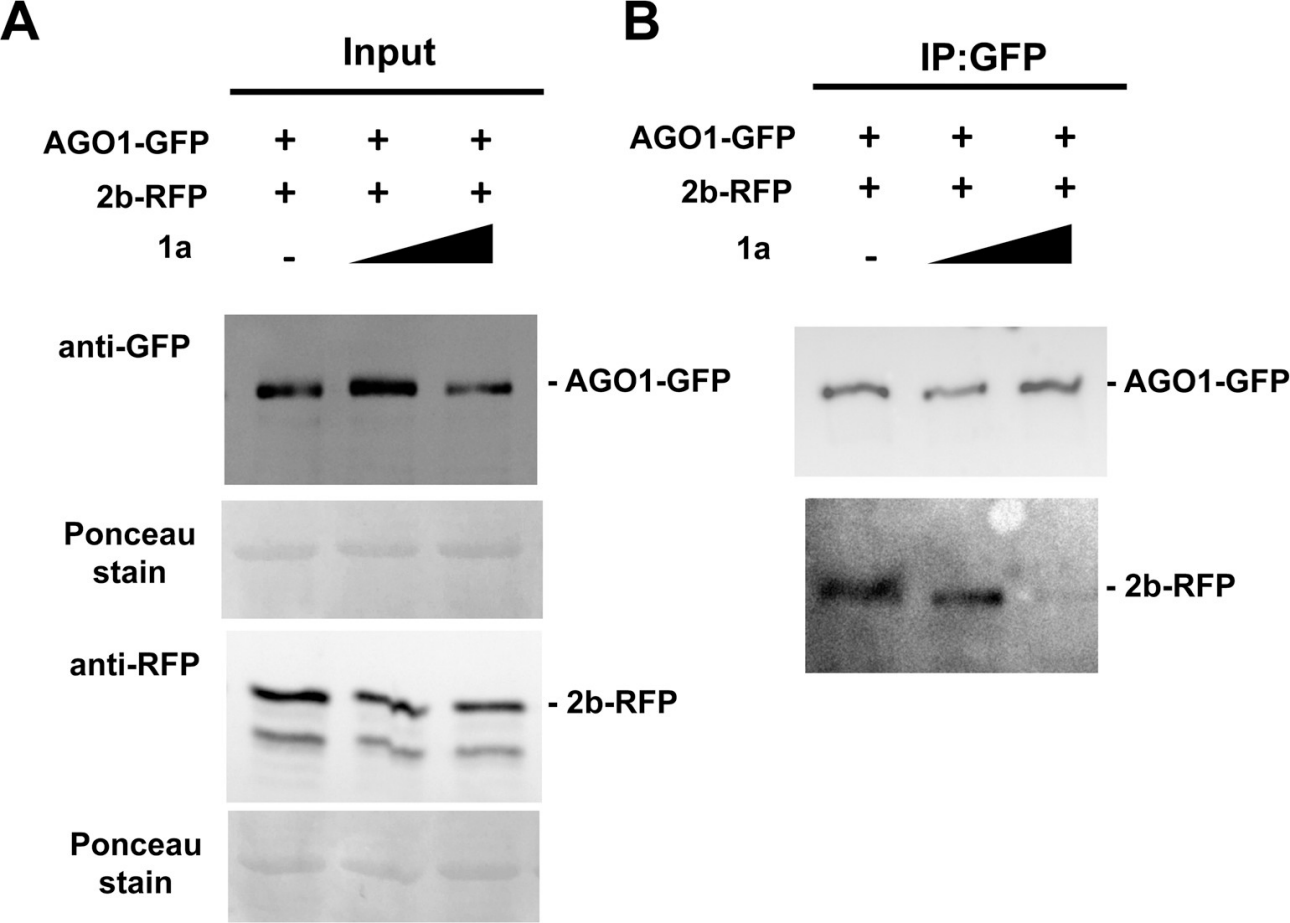
The cucumber mosaic virus 1a protein inhibits the 2b protein from binding to AGO1. A, representative western blots of AGO1-GFP and 2b-RFP extracted from *N*. *benthamiana* after transient expression. A suspension of infiltration buffer and empty Agrobacterium cells was used to dilute samples to ensure the ratio of 2b: AGO1 remained constant as increasing amounts of 1a was added. The final OD_600_ of each treatment was 1, while the relative OD_600_ of Agrobacterium expressing AGO1-GFP and 2b-RFP was 0.25 in all three treatments. The relative OD_600_ of Agrobacterium expressing 1a added was 0.25 and 0.5, which corresponded to a ratio of AGO1-GFP: 2b-RFP: 1a of 1:1:1 and 1:1:2, respectively. Total proteins were extracted and 10 ug of protein in sample buffer was loaded per well. Bottom panel shows loading control (Ponceau stain). B, representative Co-IP experiments with proteins expressed by co-agroinfiltration revealed an inhibitory effect of the CMV 1a protein on AGO1-2b interaction. The immune complexes were formed by pre-incubation with anti-GFP beads (IP AGO1-GFP) and revealed with RFP antibody (bottom panel).

The combination of co-localization and BiFC data suggests that the CMV 1a and 2b directly interact *in planta* and led us to hypothesize that this interaction limits the ability of 2b to interact with AGO1. To confirm the interaction between CMV 2b and 1a, we transiently expressed GFP-2b together with RFP-1a in *N*. *benthamiana*. Three days after agroinfiltration, GFP-2b proteins were immunoprecipitated using GFP-affinity beads and purified proteins were analyzed by western immunoblot analysis using antibodies raised against GFP or RFP (Fig 7; S4 Fig). We found that RFP-1a co-immunoprecipitated with GFP-2b, but not with the GFP-affinity beads alone. Free GFP was used as a negative control to exclude the possibility that 1a interacts non-specifically with GFP. As an additional control we tested the ability of AGO1 to interact with the 1a protein in a co-immunoprecipitation assay (S5 Fig). AGO1-GFP was unable to co-immunoprecipitate the RFP-1a protein, which is consistent with BiFC results for AGO1 and 1a that indicated that 1a and AGO1 do not interact directly *in planta* (S2 Fig). To further investigate the ability of the 1a protein to inhibit the 2b-AGO1 interaction, we carried out a competitive binding experiment. Increasing amounts of *A*. *tumefaciens* cells carrying the 1a protein sequence were co-agroinfiltrated with cells carrying 2b-RFP and AGO1-GFP coding sequences, and the ability of AGO1 to co-immunoprecipitate 2b was quantified using densitometry (Fig 8). We observed that when the 1a protein was present, AGO1 co-immunoprecipitated a smaller proportion of the 2b protein, supporting the idea that the 1a protein competes with AGO1 for interaction with the 2b protein. To further confirm if the presence of the 1a protein altered the interaction between AGO1 and the 2b protein, sYFP-tagged 2b and AGO1 constructs were co-infiltrated. We observed that addition of the 1a protein significantly reduced the intensity of fluorescence due to reconstitution of the YFP fluorophore after sYFPn-2b and sYFPc-AGO1 interaction (S3 Fig). It should be noted that there is some indication that in these assays the presence of the 2b protein may be increasing 1a protein accumulation, which is most likely explained through the stabilization of *1a* transcripts by the VSR activity of the 2b protein.

### The 1a protein alters 2b protein localization but does not affect 2b RNA silencing suppressor activity

**Fig 9:**
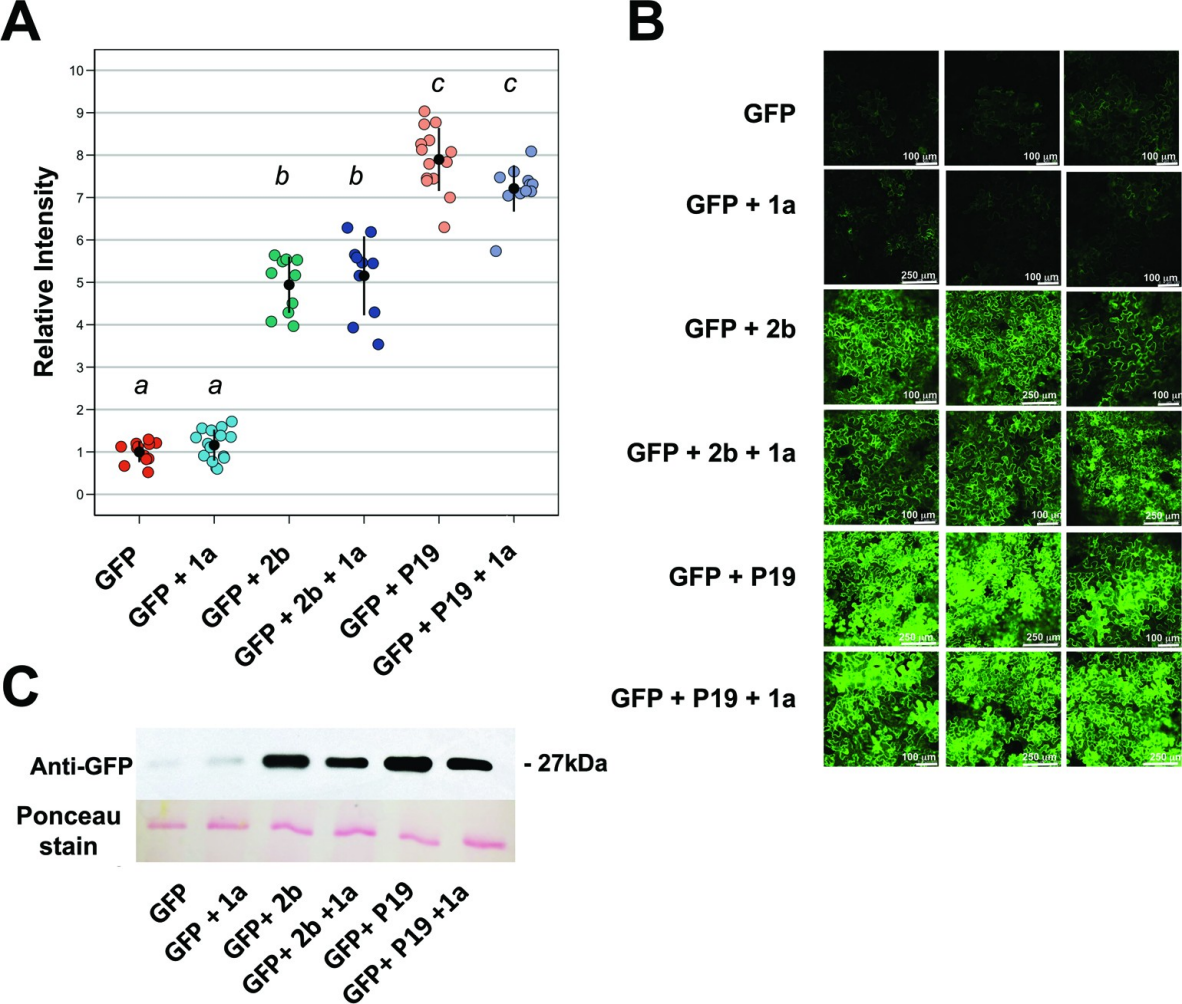
The cucumber mosaic virus 1a protein does not affect the RNA silencing suppressor activity of the 2b protein. Green fluorescent protein (GFP) was expressed transiently, under a 35S promoter, in agroinfiltrated leaves of *Nicotiana benthamiana*. A, the relative intensity of GFP fluorescence was quantified using ImageJ as the integrated density (IntDen) of each image, for each treatment 16 days after infiltration. Individual relative fluorescence values are presented as jitter plots with each mean value and standard error depicted as black bars. Compared to the intensity of fluorescence emitted by leaves expressing GFP only, the relative intensity values of GFP fluorescence emitted by leaf tissue agroinfiltrated with mixtures of *A*. *tumefaciens* cells that included those carrying constructs expressing P19 or the 2b protein were significantly greater. Lower case letters *a*, *b*, and *c* indicate mean values for relative fluorescence intensity that are significantly different from each (P <0.0001: Tukey’s multiple comparison of means). Values labeled with the same letter are not significantly different form each other. Expression of the 1a protein had no significant effect on GFP fluorescence, regardless of whether or not the 2b protein was also expressed. Number of independent leaves imaged for each treatment, n = 15. B, Typical confocal images of GFP fluorescence in the presence of 1a, 2b or P19, as indicated. When GFP-expressing *A*. *tumefaciens* was co-agroinfiltrated with CMV 2b protein or P19 protein the intensity and duration of fluorescence was increased due to their VSR activity. Co-agroinfiltration of CMV 1a protein had no effect on any of the three treatments. Number of independent leaves imaged for each treatment, n = 15. C, leaf disks where harvested 16 days after infiltration for immunoblot analysis. GFP protein accumulation was confirmed using anti-GFP antibodies.

To determine if the 1a protein inhibits the VSR activity of the 2b protein, a transiently expressed *GFP* reporter gene was agroinfiltrated into patches of *N*. *benthamiana* leaves alone or together with constructs expressing the 1a or 2b proteins (Fig 9). Following agroinfiltration, transient accumulation of free GFP fluorescence was imaged and quantified at 4, 8, and 16 days post infiltration. Agroinfiltration of the GFP construct on its own resulted in low intensity fluorescence, which decayed within a week (Fig 9). When free GFP and CMV 2b constructs were co-agroinfiltrated, both the intensity and duration of the fluorescence signal were increased (Fig 9), with GFP fluorescence visible until at least 16 days post-infiltration. P19 is the tombusvirus VSR [51], and when a P19 construct was co-agroinfiltrated this also increased the duration and intensity of the GFP signal (Fig 9).

Co-agroinfiltration of constructs encoding 1a and free GFP had no effect on the observed levels of GFP fluorescence (Fig 9), which confirmed that the 1a protein neither possesses VSR activity, nor compromises GFP stability. Since the 1a protein binds to the 2b protein, it was suspected that the presence of 1a might interfere with the VSR activity of 2b. However, co-agroinfiltration of constructs encoding 1a, 2b and free GFP did not alter the intensity or duration of fluorescence, or corresponding GFP protein levels (Fig 9). The 1a protein had no effect on the VSR activity of P19. Thus, the 1a protein does not inhibit the VSR activity of the 2b protein, and has no general anti-VSR properties.

## Discussion

### The CMV 1a protein and 2b VSR interact directly and this modulates inhibition of AGO1 activity by the 2b protein

We have shown that the CMV 1a protein has, in addition to its previously documented functions in virus replication and pathogenesis [7,10], the ability to modulate the association of the 2b VSR with one of its host targets, AGO1. AGO1 is a key target of several VSRs and inhibition of AGO1 activity for some viruses can provide an effective means of diminishing antiviral RNA silencing [13]. It was once thought that cucumoviral 2b VSRs inhibit antiviral RNA silencing by binding to AGO1 [30] until subsequent work showed that their VSR activity is actually dependent upon the ability to titrate double-stranded siRNAs [23–28]. In Arabidopsis, inhibiting AGO1 activity may be a counterproductive means of inhibiting antiviral RNA silencing. AGO1 regulates *AGO2* mRNA levels using miR403 and de-repression of AGO2 accumulation by the 2b protein triggers the establishment of another layer of antiviral silencing [52]. Additionally, inhibition of AGO1 activity by the 2b protein induces antibiosis against the aphid vectors of CMV [41]. Thus, in Arabidopsis the CMV 1a protein plays an important role in preventing the 2b protein from triggering these additional lines of host defense against the virus and its vector.

In tobacco, by contrast, it appears that the CMV 1a protein can trigger antibiosis against aphids; an effect counteracted by the 2b protein [40,46]. In both of these hosts, Arabidopsis and tobacco, the 1a and 2b proteins have antagonistic roles in conditioning CMV-induced effects on aphid-plant interactions suggesting the interplay of the 1a and 2b proteins determines the effect of CMV infection on plant-aphid interactions in different hosts, i.e., induction of aphid resistance, or of aphid susceptibility. This reinforces previous work showing that the effects of viral proteins on plant-aphid interactions are complex and combinatorial [18,41,46].

In *2b*-transgenic Arabidopsis plants the 2b protein induces stunting of shoots and roots, and developmental abnormalities, including floral deformation [32]. These effects occur in large part through inhibition of AGO1 activity, in particular, by inhibition of mRNA slicing directed by miR159 [22,31–33]. The symptom-like phenotypes of *2b*-transgenic plants can be exaggerated compared with the symptoms seen in CMV-infected, non-transgenic plants [32]. We think it likely that by binding the 2b protein and ameliorating these 2b-induced phenotypes, the 1a protein may limit the deleterious effects of CMV infection on the host. This would be beneficial for CMV since excessive damage to the host plant may diminish virus yield, or decrease the period of time during which the plant is infectious (e.g. by decreasing its lifespan), or inhibit the ability of susceptible hosts to reproduce, which would favor the emergence of resistant individuals in the host population, an effect modeled by Groen et al. [53].

Modulating 2b activity would benefit aphid-mediated CMV transmission. In Arabidopsis, AGO1 negatively regulates antibiosis against aphids [41,47], and 2b-induced inhibition of AGO1 activity, as seen in *2b*-transgenic plants, is deleterious to aphids and would compromise their ability to vector the virus. Our data confirm that 1a prevents induction of antibiosis by the 2b protein in Arabidopsis and they suggest a mechanism by which direct interaction between 1a and 2b, will regulate the extent of 2b-mediated inhibition of AGO1 (Fig 9).

### The CMV 1a protein associates with P-bodies

As is common for plus-strand RNA viruses, CMV RNA replication occurs in close association with intracellular membranes [7,54]. Previous studies using electron microscopy and immunogold detection, and cellular fractionation localized the CMV replicase complex to the vacuolar membrane (tonoplast) in tobacco and cucumber [4]. In contrast, the 2a protein was observed in cytoplasmic and membrane-associated fractions [8]. However, there have been few recent investigations of the subcellular localization of the CMV 1a and 2a proteins. When expressed transiently in *N*. *benthamiana* we observed a distinct subcellular localization pattern for the 1a protein of punctate ‘specks’ throughout the cytoplasm. We therefore hypothesized that the CMV 1a protein may localize to ER-derived vesicular replication structures equivalent to those reported for the orthologous BMV 1a protein [55]. The BMV 1a protein is known to remodel ER membrane morphology and permeability to promote viral replication [56]. However, ER staining did not associate with the ‘specks’ indicating that the CMV 1a does not associate with the ER.

P-bodies play roles in mediating mRNA decapping and decay, and in miRNA-induced RNA slicing, and their formation is increased as a consequence of RNA silencing [57,58]. We observed that the P-body marker DCP1 co-localized with the CMV 1a protein in the punctate specks, indicating that the 1a protein associates with P-bodies. A proportion of 2b protein was recruited to the P-bodies by binding to the 1a protein, although 2b protein was still present in its nuclear and cytoplasmic locations. It is possible that by re-localizing the 2b protein to P-bodies, the 1a protein limits the ability of the 2b protein to interact with AGO1 and inhibit AGO1-mediated miRNA-directed slicing activity.

The P-body associated complex LSm1-7 [59] functions in mRNA decapping within in the 5′–3′ exoribonucleolytic pathway [60]. The LSm1-7 complex regulates BMV RNA translation and viral replication [61]. In yeast, it was shown that *cis*-acting sequences on replicons derived from BMV RNAs 2 and 3 were found to direct these molecules into P-bodies [48]. A population of P-bodies associate with membranes where BMV and other positive-sense viruses replicate, and it was suggested that P-bodies play a role in the transition of BMV RNAs from serving as translation templates to acting as replication templates. The interaction of P-bodies containing viral proteins with membranes could facilitate interactions between the membrane-bound BMV 1a protein, and the components in P-bodies, thereby leading to the assembly of the replication complex [48,61,62]. We suspect that it is possible that P-bodies also play a role in CMV replicase assembly.

### The interaction of 1a and 2b does not inhibit 2b VSR activity

The 2b protein performs its VSR role primarily in the cytoplasm [25]. Increasing the nuclear and nucleolar enrichment of Fny-2b compromises its VSR activity but enhances CMV virulence, accelerating the appearance of disease symptoms in Arabidopsis plants [22]. Similar to CMV 2b, other VSRs, including the potyviral HC-Pro and tombusviral P19, bind sRNAs [63–65] and are most effective as inhibitors of antiviral RNA silencing when present in the cytoplasm [66,67]. For example, translocation of P19 into the nucleus by host ALY proteins greatly impairs its VSR activity, demonstrating that binding sRNAs by P19 occurs in the cytoplasm [68]. Other host proteins can inhibit VSR activity. For example, the tobacco rgsCAM protein binds to VSRs of several viruses, including the CMV 2b protein; inhibiting and destabilizing them [69].

There are also indirect effects of certain viral proteins on VSR activity. For example, the catalytic activity of the P1 protease of the potyvirus plum pox virus governs the release of the HC-Pro VSR during polyprotein processing [70]. In the pepo strain of CMV it was shown that an arginine-rich domain of the coat protein inhibited translation of viral RNA, which limited 2b protein accumulation [71]. However, to our knowledge the inhibition of just one of the effects of the 2b protein on the host RNA silencing network (i.e., inhibition of AGO1 activity) is the first documented instance of regulation of one specific function of a VSR by direct interaction with another viral protein.

Although the 1a protein directly interacts with the 2b protein, alters its localization, and inhibits the AGO1-2b protein interaction, it has no effect on 2b VSR activity. The results are consistent with our previous work showing that 2b-mediated inhibition of antiviral RNA silencing, and 2b-mediated inhibition of AGO1-mediated, miRNA-directed mRNA cleavage are separate 2b functions and determined by different functional domains within the 2b protein [23,25]. Our data suggests that re-localization to P-bodies by the 1a protein does not diminish the ability of 2b to inhibit RNA silencing. Furthermore, it shows that the 1a protein is able to inhibit the induction of 2b-induced antibiosis against aphids and ameliorate 2b-mediated disruption of plant development without disrupting the ability of the 2b protein to perform its vital counter-defense role.

## Conclusions

**Fig 10:**
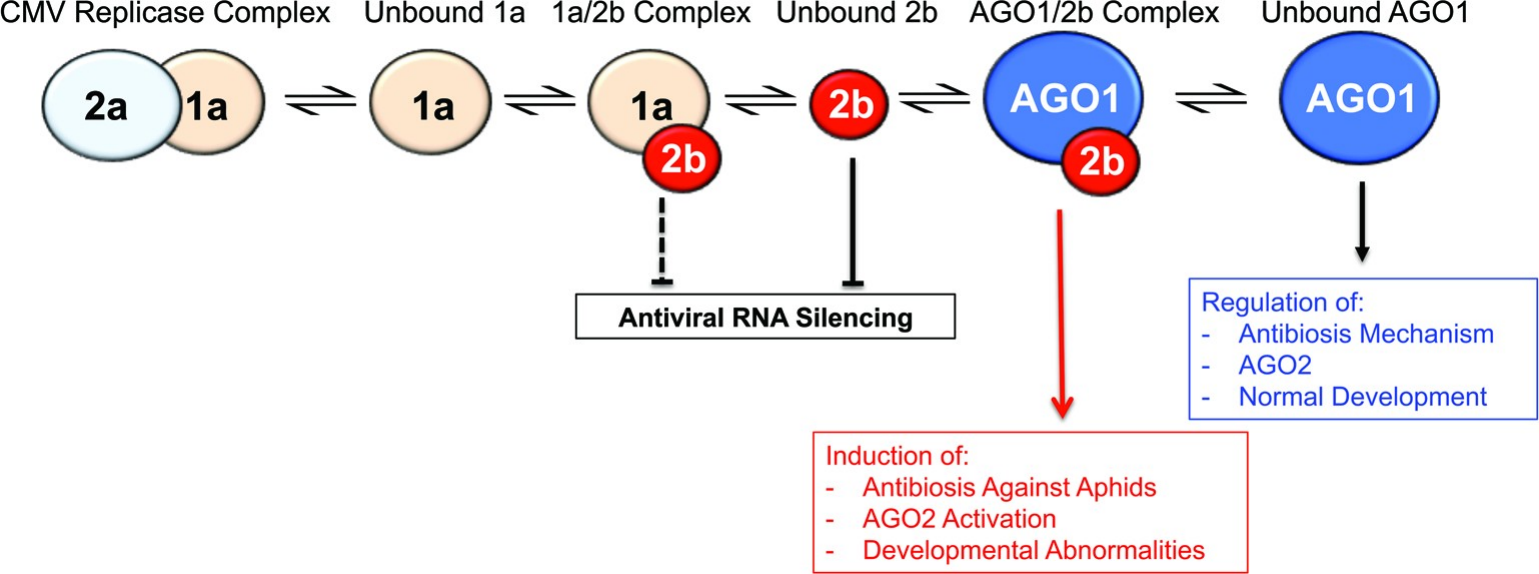
Interaction between the cucumber mosaic 1a and 2b proteins regulates the 2b-mediated inhibition of AGO1 activity. The primary function of the cucumber mosaic virus (CMV) 1a protein is, together with the 2a RNA-dependent RNA polymerase protein, to function as a virally encoded component of the CMV replicase complex [7]. In this study we have shown that 1a protein can also bind to the CMV 2b suppressor of RNA silencing. This does not affect the ability of the 2b protein to inhibit antiviral RNA silencing. Most 2b protein is not bound to 1a (Fig 4), and so its ability to inhibit antiviral silencing is not suppressed (blunt arrow). We have no evidence that the 1a-2b interaction inhibits the ability of the bound 2b to suppress antiviral silencing but at this time it cannot be ruled out (hence the blunt arrow is dashed in this diagram). Under the direction of microRNAs miR403 and miR159, respectively, AGO1 regulates the accumulation of *AGO2* mRNA, and mRNAs encoding host developmental regulators [31,52]. When the 2b protein binds to AGO1 this system is de-regulated, leading to induction of developmental abnormalities (disease symptoms) [31], and increasing the accumulation of AGO2, which triggers an additional layer of resistance to CMV [52]. It also releases negative regulation of antibiosis against aphids, the insect vectors of CMV [41]. We hypothesize that the 1a-2b interaction moderates the inhibitory effect of the 2b protein on AGO1. Key: blunt arrows indicate negative regulation or inhibition; the red arrow indicates activation/de-repression of AGO1-regulated processes, and the black arrow normal functioning of AGO1.

We conclude that the CMV 1a protein directly interacts with the 2b protein and this inhibits the 2b-AGO1 interaction. The results suggest that the interaction of the 1a and 2b proteins does not negate the VSR activity of the 2b protein. Another possibility is that 2b protein molecules bound to 1a protein molecules are not able to bind small RNAs (Fig 10) but that sufficient unbound 2b is present to maintain antiviral silencing. This possibility is also consistent with our data, which indicate that a relatively small proportion of the overall 2b protein pool binds to the 1a protein (see Fig 4). However, this will need to be investigated in future work by determining, among other things, whether or not the sequences of the 2b protein required for silencing suppression are distinct from those required for interaction with the 1a protein. This suggests that 1a negatively regulates the inhibition of AGO1 by the 2b protein, which may ameliorate the potential damage caused by CMV to its hosts, and in Arabidopsis would prevent the induction of strong resistance (antibiosis) against its aphid vectors, while allowing 2a-induced feeding deterrence (antixenosis: which benefits virus transmission) to predominate (Fig 10). The effect of 1a on the 2b protein, however, does not affect its ability to suppress RNA silencing and so is also beneficial to the virus. Furthermore, moderating the inhibition of AGO1 further benefits the virus because it prevents induction of another layer of antiviral RNA silencing that is mediated by AGO2 [52]. Thus, the interaction between the 1a replication protein and the 2b VSR represents a novel form of regulation by which a virus is able to modulate its ability to induce symptoms and suppress host resistance while simultaneously modifying interactions between its host and its insect vectors.

## Material and methods

### Plants

*Nicotiana benthamiana* Domin. seeds were germinated on moist soil. After one week, seedlings were transferred to pots containing Levington M3 compost (Scotts, Surrey UK) in a Conviron (Manitoba, Canada) growth room maintained at 22°C, 60% relative humidity and 200 μmol m^-2^ s^-1^ photosynthetically-active radiation under a 16 h light/ 8h dark light regime. *Arabidopsis thaliana* (L.) Heynh. accession Col-0 plants used in this study were grown at 20°C and 70% relative humidity under short-day conditions (8 h light/16 h dark). The *2b*-transgenic Arabidopsis lines 3.13F and 2.30F were constructed by Lewsey et al. [32] and transgenic plants constitutively expressing the CMV 1a protein and double *1a/2b*-transgenic plants were previously described by Westwood et al. [41].

### Aphid experiments

Aphids (*Myzus persicae* Sulzer clone US1L [72]) were maintained on *Brassica rapa pekinensis* plants. To obtain aphids of a standardized developmental stage for use in experiments, reproducing adults were transferred to un-infested *B*. *rapa*, allowed to reproduce for no longer than 24 hours and the resulting one-day-old nymphs were transferred singly to 4-week-old Arabidopsis plants using fine paintbrushes and confined on plants using micro-perforated plastic bags (Associated Packaging, Kent, UK) [41]. Colony size was recorded 10 days later [40].

### DNA constructs

Viral sequences used in this work were derived from the Fny strain of CMV [73]. The pROK2-based vectors for bimolecular fluorescence complementation (BiFC) were generated by amplifying the N- and C-terminal domains from the yellow fluorescent protein (YFP) ORF, which were cloned into the *Xba*I and *Bam*HI linearized pROK2 vector [74]. This insertion left a BamHI-XmaI-KpnI-SacI polylinker downstream of the inserted N- and C-terminal halves of the YFP sequence into which the Fny 1a ORF was amplified using appropriate primers (S1 Table). These fused ORFs were cloned into the BamHI and XmaI digested pROK-sYFP backbone to generate the pROK constructs sYFPn-1a and sYFPc-1a. For BiFC analysis pROK constructs expressing sYFPn-2a and sYFPc-2a were also generated. Additional pROK constructs expressing sYFPn-2b, sYFPc-2b, sYFPn-AGO1 and sYFPc-AGO1 were previously described by González et al. [23]. *GFP* and *mRFP* sequences were amplified to introduce BamHI and ApaI overhangs (S1 Table) and then cloned into the BamHI and ApaI digested 1a-pMDC32 vector to generate, respectively, the GFP:1a-pMDC32 and mRFP:1a-pMDC33 constructs. This approach followed work by Westwood et al. [41], who previously constructed the pMDC32 construct expressing untagged Fny 1a protein. DCP1-GFP and RFP-DCP1 constructs were constructed using Gateway cloning. Initially, the Arabidopsis DCP1 ORF was amplified from Arabidopsis cDNA using forward and reverse primers that contained 5’ extensions corresponding to the *att*B site (S1 Table). The purified PCR products containing *att*B sites were then cloned into the pDONR221 (Invitrogen) vector by a one-hour incubation with Gateway BP clonase at 25°C. The verified entry clones were then combined with pSITE-2NB or pSITE-4CA vectors [75] in an overnight LR clonase recombination reaction to produce DCP1-GFP and RFP-DCP1, respectively. Additional DCP1-RFP and DCP2-RFP N-terminal fusion constructs were generously provided by Dr Nina Lukhovitskaya [76].

### Agroinfiltration

*Agrobacterium tumefaciens* (GV3101) cells were grown at 28°C with shaking in 50 ml of liquid LB medium [77] containing the appropriate vector antibiotic and 50 μg/ml rifampicin and 10 μg/ml gentamicin. This was then incubated at 28°C overnight. Cultures were centrifuged for 15 min at 5000 *g* and re-suspended in 10 mM MgCl_2_, 10 mM MES pH 5.6, and 100 μM acetosyringone. Each suspension was adjusted to an OD_600_ of 0.5 (unless stated otherwise) to ensure the same number of cells bearing each construct was infiltrated. Cell suspensions were incubated at room temperature for 2 hours prior to agroinfiltration, using the blunt end of a syringe, into the abaxial surface of leaves of 3-week-old *N*. *benthamiana* plants.

### RNA silencing suppression assays

A free GFP reporter was expressed transiently from a binary vector under the control of the CaMV 35S promoter in a *N*. *benthamiana* leaf by agroinfiltration, either by itself or with another vector expressing a protein to be tested for suppression of silencing activity [23]. To monitor the effect of a second protein on the levels of fluorescence derived from the transiently expressed free GFP, leaves were imaged 4, 8 and 16 days after agroinfiltration using a Leica SP5 confocal microscope. At 16 days after infiltration a cork borer was used to excise tissue from the infiltrated zone, which was flash-frozen in liquid N_2_ for subsequent protein extraction and immunoblot analysis.

### Confocal laser scanning microscopy and histochemical staining

All confocal microscopy was performed on a Leica Model SP5. GFP was excited using 488 nm laser lines, RFP at 561 nm and YFP at 514 nm. Staining of ER was achieved with ER-tracker (Invitrogen). A concentration of 1 μM was prepared in phosphate buffer and infiltrated into *N*. *benthamiana*. Dye was left for 30 min before infiltrating the patch with phosphate buffer to flush excess dye. Leaf sections were imaged using excitation and emission maxima at 587 nm and 615 nm, respectively. The styryl dye FM 4–64 (Invitrogen) was infiltrated into the abaxial surface of *N*. *benthamiana leaves* at 25 mM in sterile water. Images were taken 1 hour after infiltration. FM 4–64 was imaged using an excitation and emission maxima at 515 nm and 640 nm, respectively.

### Western immunoblot analysis

Proteins were extracted from agroinfiltrated leaf tissue by homogenization in protein extraction buffer [10% glycerol, 25 mM Tris-HCl (pH 7.5), 200 mM NaCl, 1 mM ethylenediaminotetraacetic acid (EDTA), 0.15% IGEPAL CA-630 (Octylphenoxy poly(ethyleneoxy)ethanol, branched) (Merck), 2% PVP-40, 10 mM dithiothreitol, and protease inhibitor cocktail]. Proteins were analyzed by SDS-PAGE [78] and electrophoretically transferred onto nitrocellulose membranes [79]. Anti-GFP (1:1000) and anti-RFP (1:2000) monoclonal antibodies (Chromotek) were used to detect accumulation of GFP and RFP, respectively. Anti-rabbit or anti-mouse IgG horseradish peroxidase conjugated secondary antibodies were diluted to 1:10,000 for use. Bands were visualized by incubating membranes with Pierce Enhanced Chemiluminescence ECL-Plus substrate and exposure on X-ray film.

### Co-Immunoprecipitation assays to detect *in vivo* protein-protein interactions

Wild-type *N*. *benthamiana* leaves were infiltrated with *Agrobacterium tumefaciens* and at 4 dpi, leaves were collected and pulverized in liquid nitrogen, and proteins were extracted in extraction buffer [10% glycerol, 25mM Tris-HCL (pH 7.5), 1 mM EDTA, 150 mM NaCL, 10 mM dithiothreitol, protease inhibitor cocktail (Roche), 1 mM phenymethylsulfonylfluoride]. The crude extract was pelleted by centrifugation for 4 min at 12,000 *g* at 4°C, and the supernatant collected. The supernatant was subjected to immunoprecipitation by adding 25 μl of equilibrated GFP-Trap agarose beads (Chromotek) to 300 μl of supernatant and 300 μl of dilution buffer [10 mM Tris-HCl (pH 7.5),150 mM NaCl, 0.5 mM EDTA], and placed on a rotary incubator for 1 h at 4°C. The GFP-Trap agarose beads were washed three times with dilution buffer. The GFP-Trap agarose beads were collected by centrifugation at 2500 *g* for 2 min before being re-suspended in 100 μl 2 x SDS-sample buffer [78] and heated for 10 min at 95°C to dissociate immunocomplexes from the beads. After centrifugation at 2500 *g* for 2 min at 4°C, the supernatant was analyzed by SDS-PAGE.

## Supporting information

S1 Table Primers used in cloning of fusion proteins. (DOCX)

S1 Fig The 1a protein does not alter the localization of free GFP. When expressed in *N*. *benthamiana* GFP accumulates in the cytoplasm. When RFP-1a and 35S:GFP were co-agroinfiltrated we did not observe a change in either proteins localization suggesting that the unspecific binding of GFP to the 1a protein does not occur.
(TIFF)

S2 Fig The cucumber mosaic virus 1a protein interacts with itself and the 2a protein but not with AGO1. A, when split (s)YFPn-1a and sYFPc-1a where co-expressed we observed foci of faint fluorescence. Self-interaction of the 1a protein has previously been reported (O’Reilly et al. 1998). B, we observed small foci of fluorescence when sYFP-1a and sYFP-2a where co-expressed, this was expected as these proteins form the viral replicase. C, D, when sYFP-1a and sYFP-AGO1 where co-expressed no fluorescence was observed suggesting that these proteins do not interact *in vivo*.
(TIFF)

S3 Fig There is decreased fluorescence from the *in planta* interaction between sYFPn-2b and sYFPc-AGO1 in the presence of the cucumber mosaic virus 1a protein. Agro-cultures of sYFPn-AGO1 and sYFPc-2b were coinfiltrated into *N*. *benthamiana* leaves at a final OD_600_ of 0.9. Untransformed Agrobacterium (GV3101) cells resuspended in infiltration buffer were used to prepare the final OD_600_ so that the relative OD_600_ of each construct was 0.3. The RFP-1a construct was coinfiltrated with sYFPn-AGO1 and sYFPc-2b at a ratio of 1:1:1 with a final OD_600_ of 0.9. The intensity of YFP fluorescence for each image was calculated using the Lecia Application Suite X (LAS X). Measurements were collected from 5 individual plants, that were each infiltrated at 5 patches giving a total of 25 images for each treatment. Asterisks indicate significant difference [Student’s t-test *, P<0.05; **, P<0.01; ***, P<0.001]. Error bars represent standard error of the mean.
(TIFF)

S4 Fig Association of the 1a and 2b proteins *in planta* demonstrated by co-immunoprecipitation. A, total proteins expressed in *N*. *benthamiana* leaves were subjected to immunoprecipitation with GFP-Trap beads followed by immunoblot analysis with anti-GFP antibodies to detect GFP-2b or 35S:GFP and anti-RFP antibodies to detect RFP-1a. RFP-1a could be detected in both input samples with a corresponding band of approximately 138kDa. After Immunoprecipitation with GFP-pull down RFP-1a could only be detected when co-expressed with GFP-2b, and was not detected when expressed with 35S:GFP. B, as the band corresponding to RFP-1a in the IP:GFP sample was relatively faint the blot was exposed for 10 and 30 minutes to ensure RFP-1a wasn’t carried through when co-expressed with GFP. Black rectangles indicate bands used to form the composite blot in Fig 6C, the loading control is shown for the input sample stained with Ponceau stain.
(TIFF)

S5 Fig AGO1 does not bind to the cucumber mosaic virus 1a protein *in planta*. Total proteins purified from agroinfiltrated *N*. *benthamiana* leaves were subjected to immunoprecipitation with RFP-Trap beads followed by immunoblot analysis with anti-GFP antibodies to detect AGO1-GFP and anti-RFP antibodies to detect RFP-1a or 2b-RFP. AGO1-GFP could be detected in both input samples with a corresponding band of approximately 140kDa. After Immunoprecipitation with RFP-Trap AGO1-GFP could only be detected when co-expressed with 2b-RFP, and was not detected when expressed with RFP-1a. RFP-1a and 2b-RFP were both detected after immunoprecipitation with RFP-Trap beads. The loading control is shown for the input sample stained with Ponceau stain.
(TIFF)
