# Supplementary material for: The cucumber mosaic virus 1a protein regulates interactions between the 2b protein and ARGONAUTE 1 while maintaining the silencing suppressor activity of the 2b protein
Source: PLoS Pathog. 2020 Dec 3;16(12):e1009125. doi: 10.1371/journal.ppat.1009125 (PMC7738167; doi:10.1371/journal.ppat.1009125)
Supplement: S1 Table — (DOCX) [file ppat.1009125.s001.docx]

**S1 Table. Primers used in cloning of fusion proteins.**

| **Primer name** | **Sequence 5’-3’** |
| --- | --- |
| mRFP-BamHI-Fw | GGGCCCGGATCCATGGCCTCCTCCGAGGAC |
| mRFP-ApaI-Rv | GGCGCGCCGGGCCCAGGCGCCGGTGGAGTG |
| GFP-BamH1-Fw | TAGGGCCCGGGATCCTGATGGTGAGCAAGGGCGAG |
| GFP-ApaI-Rv | GATCCCGGGCCCTATACTTGTACAGCTCGTCCAT |
| 1a-BamHI-Fw | CTGCTAGGATCCATGGCGACGTCCTCGTTCAACATC |
| 1a-XmaI-Rv | ATCTAGCCCGGGCTAAGCACGAGCAACACATT |
| DCP1-att-Fw | GGGGACAAGTTTGTACAAAAAAGCAGGCTTAATGTCTCAAAACGGGAAGA  TAATCCCA |
| DCP1-att-Rv | GGGGACCACTTTGTACAAGAAAGCTGGGTTTTATTGTTGAAGTGCATTT  TGTAAAGTTCGG |
| DCP1-Cterm-RFP-att-Rv | GGGGACCACTTTGTACAAGAAAGCTGGGTGTTGTTGAAGTGCATTTTGTA  AAGTTCGG |
