# Supplementary figures and images for: The cucumber mosaic virus 1a protein regulates interactions between the 2b protein and ARGONAUTE 1 while maintaining the silencing suppressor activity of the 2b protein
Source: PLoS Pathog. 2020 Dec 3;16(12):e1009125. doi: 10.1371/journal.ppat.1009125 (PMC7738167; doi:10.1371/journal.ppat.1009125)

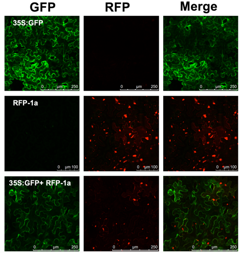

Supplement: S1 Fig — When expressed in N. benthamiana GFP accumulates in the cytoplasm. When RFP-1a and 35S:GFP were co-agroinfiltrated we did not observe a change in either proteins localization suggesting that the unspecific binding of GFP to the 1a protein does not occur. (TIFF) [file ppat.1009125.s002.tiff]

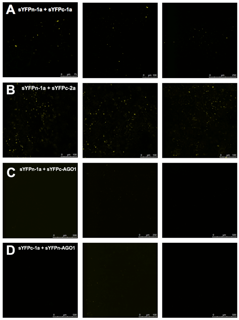

Supplement: S2 Fig — A, when split (s)YFPn-1a and sYFPc-1a where co-expressed we observed foci of faint fluorescence. Self-interaction of the 1a protein has previously been reported (O’Reilly et al. 1998). B, we observed small foci of fluorescence when sYFP-1a and sYFP-2a where co-expressed, this was expected as these proteins form the viral replicase. C, D, when sYFP-1a and sYFP-AGO1 where co-expressed no fluorescence was observed suggesting that these proteins do not interact in vivo. (TIFF) [file ppat.1009125.s003.tiff]

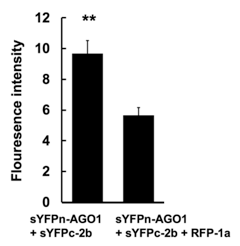

Supplement: S3 Fig — Agro-cultures of sYFPn-AGO1 and sYFPc-2b were coinfiltrated into N. benthamiana leaves at a final OD600 of 0.9. Untransformed Agrobacterium (GV3101) cells resuspended in infiltration buffer were used to prepare the final OD600 so that the relative OD600 of each construct was 0.3. The RFP-1a construct was coinfiltrated with sYFPn-AGO1 and sYFPc-2b at a ratio of 1:1:1 with a final OD600 of 0.9. The intensity of YFP fluorescence for each image was calculated using the Lecia Application Suite X (LAS X). Measurements were collected from 5 individual plants, that were each infiltrated at 5 patches giving a total of 25 images for each treatment. Asterisks indicate significant difference [Student’s t-test *, P<0.05; **, P<0.01; ***, P<0.001]. Error bars represent standard error of the mean. (TIFF) [file ppat.1009125.s004.tiff]

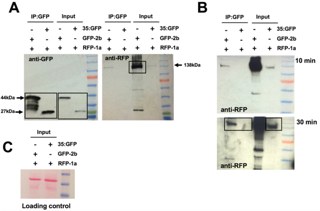

Supplement: S4 Fig — A, total proteins expressed in N. benthamiana leaves were subjected to immunoprecipitation with GFP-Trap beads followed by immunoblot analysis with anti-GFP antibodies to detect GFP-2b or 35S:GFP and anti-RFP antibodies to detect RFP-1a. RFP-1a could be detected in both input samples with a corresponding band of approximately 138kDa. After Immunoprecipitation with GFP-pull down RFP-1a could only be detected when co-expressed with GFP-2b, and was not detected when expressed with 35S:GFP. B, as the band corresponding to RFP-1a in the IP:GFP sample was relatively faint the blot was exposed for 10 and 30 minutes to ensure RFP-1a wasn’t carried through when co-expressed with GFP. Black rectangles indicate bands used to form the composite blot in Fig 6C, the loading control is shown for the input sample stained with Ponceau stain. (TIFF) [file ppat.1009125.s005.tiff]

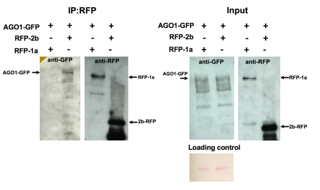

Supplement: S5 Fig — Total proteins purified from agroinfiltrated N. benthamiana leaves were subjected to immunoprecipitation with RFP-Trap beads followed by immunoblot analysis with anti-GFP antibodies to detect AGO1-GFP and anti-RFP antibodies to detect RFP-1a or 2b-RFP. AGO1-GFP could be detected in both input samples with a corresponding band of approximately 140kDa. After Immunoprecipitation with RFP-Trap AGO1-GFP could only be detected when co-expressed with 2b-RFP, and was not detected when expressed with RFP-1a. RFP-1a and 2b-RFP were both detected after immunoprecipitation with RFP-Trap beads. The loading control is shown for the input sample stained with Ponceau stain. (TIFF) [file ppat.1009125.s006.tiff]
